# Neutrophil and Macrophage Response in Acinetobacter Baumannii Infection and Their Relationship to Lung Injury

**DOI:** 10.3389/fcimb.2022.890511

**Published:** 2022-07-06

**Authors:** Zhaojun Liu, Wei Xu

**Affiliations:** Department of Pediatrics, Shengjing Hospital of China Medical University, Shenyang, China

**Keywords:** acinetobacter baumannii, macrophages, neutrophils, innate immunity, lung injury, immunotherapy

## Abstract

Acinetobacter baumannii (AB) infection has become a threat to global public health. AB is one of the main pathogens causing nosocomial infections, especially ventilator-associated pneumonia. AB easily develops resistance against a variety of antibiotics, which makes the treatment of AB infections difficult. Therefore, it is necessary to study new treatment plans like anti-infection immunity. Both animal models of AB infection and *in vitro* cell experiments show that macrophages are activated in the early stage of the immune response and regulate the recruitment of neutrophils, thus playing a role in clearing AB. AB components and the immune responses they induce can lead to injury of the infected organ, mostly in the lungs. Understanding the response of innate immunity to ABs at different stages after infection and the relationship between the response and lung injury can help to develop new immunotherapy methods and prevent lung injury. This article provides a comprehensive review of the response of neutrophils and macrophages to AB infection and their association with lung injury to develop effective therapies for AB infection and prevent lung injury.

## Introduction

Acinetobacter baumannii (AB) is one of the main causes of nosocomial infection. It is a Gram-negative bacterium prone to multidrug resistance (MDR) (Munier et al., 2019; Yadegarynia et al., 2013). AB infection can manifest in a variety of diseases of which the most common is ventilator-associated pneumonia (VAP). Other diseases such as catheter-related blood and urinary tract infection, sepsis or bacteremia, meningitis, and burn and wound-related skin and soft tissue infections are also not uncommon (Dijkshoorn et al., 2007; Chen, 2020). Bacterial components of AB, including outer membrane protein A (OmpA), outer membrane vesicles (OMVs), and lipopolysaccharide (LPS), are involved in the pathogenesis and induction of the host immune response during infection. AB exhibits significant genetic plasticity and natural genetic transformation contributes to the acquisition of genetic elements, which causes antibiotic resistance in AB and causes it to evolve and pass it on from generation to generation (Pires and Parker, 2019; Gu et al., 2021). Currently, the treatment of MDR ABs is mainly based on the combination of two or more antibiotics. There are few powerful and effective antibiotics for MDR strains under development, but progress in the development of new intervention measures is limited, which makes the treatment of MDR AB infection increasingly difficult. In addition to the use of antibiotics, appropriate immune participation in the human body also plays a vital role in bacterial clearance (Liu et al., 2020). In recent years, there have been multiple studies on the relationship between immunity and AB infections. These studies (Pires and Parker, 2019; Chen, 2020) show that innate immune cells, especially macrophages and neutrophils, play an important role in controlling early AB infection as important bacterial cleaning tools and can be useful in immunogenic treatment for AB infection in the future. Eliminating bacteria before serious organ damage is a major protection for the human body. Therefore, it is important to study early innate immunity.

The current immunotherapy strategy is to induce immune memory by stimulating adaptive immunity to establish a rapid and specific immune response after real infection. This method requires multiple vaccine injections every few weeks or months (Gu et al., 2021). However, the window of AB infection in hospitalized patients cannot meet the time required by the adaptive immunity strategy. In other words, existing immunotherapy cannot produce rapid and sufficient protection for infected patients. Therefore, the study of the early successful immune response after AB infection provides a key basis for timing and methods of artificial immune intervention. Innate immunity has, thus, become a target for new therapeutic measures (Gu et al., 2021). This article reviews the changes and mechanisms of macrophages and neutrophils in response to AB infection, combined with the progress of lung injury in the interaction between infection and immunity, to take appropriate immune measures, control infection early, reduce lung injury, and improve prognosis.

## Response and Mechanism of Macrophages in AB Infection

Macrophages reside in various tissues and are the frontline cells of innate immunity. Stimulated by different tissue microenvironments, macrophages polarize and produce M1 and M2 subtypes whose functions differ. M1 macrophages are responsible for antibacterial activity, producing pro-inflammatory cytokines, and mediating tissue damage, whereas M2 macrophages produce anti-inflammatory cytokines and promote tissue repair (Shapouri-Moghaddam et al., 2018). In mouse models, before infection, the cells in mouse ascites are mainly macrophages, and nearly all cells in bronchoalveolar lavage fluid (BALF) are composed of macrophages (Qiu et al., 2012; Harris et al., 2019; Zeng et al., 2019). In the earliest stage of AB infection, both LPS of AB and TNF-α produced by pulmonary epithelium interacted with AB can induce M1 macrophages to activate from resting M1 macrophages (Bist et al., 2014; Shapouri-Moghaddam et al., 2018; Murray and Wynn, 2011). LPS activates innate immunity by interacting with macrophage Toll-like receptor 4 (TLR4), resulting in M1 macrophages producing a large number of proinflammatory cytokines and chemokines, which induce the recruitment of macrophages and neutrophils from bloodstream and nearby tissues to the infection site. Prior to the neutrophil recruitment, macrophages play a role in clearing ABs in the early stages of infection by producing nitric oxide and reactive oxygen species (ROS). In a mouse model of AB infection delivered the intraperitoneal or respiratory tract, the number of macrophages did not change significantly within 24 h after infection, but the number of neutrophils gradually increased, accompanied by a significant decrease in the bacterial load in each organ (Qiu et al., 2012; Harris et al., 2019; Zeng et al., 2019). Relevant studies (Qiu et al., 2012; Lazaro-Diez et al., 2017) have shown that macrophages only play a moderate role in the phagocytosis and killing of ABs. In order to explore the ability of macrophages to phagocytize and kill AB, Lazaro-Diez et al. (Lazaro-Diez et al., 2017) carried out an infection experiment using a mixed culture containing human neutrophils and differentiated macrophages. It was found that the incubation of AB with macrophages and neutrophils did not induce obvious phagocytosis of macrophages, but the shape of macrophages was elongated. This suggests that AB infection can significantly activate the macrophages. Qiu et al. (Qiu et al., 2012) showed that the number of bacteria killed by macrophages after phagocytosis of AB was time-dependent, with the number of bacteria killed by macrophages 48 h after phagocytosis of ABs reaching 99%. However, compared with more than 90% of bacteria killed by neutrophils one hour after infection and phagocytosis of ABs, macrophages are far less efficient in killing ABs. Moreover, with the evolution of MDR AB, its gene will undergo some changes to adapt to various environmental stress conditions, such as H_2_O_2_ in macrophages. Studies have shown that there are inserted sequence elements and new transcription factors in *katG and katE*, the genes encoding catalase peroxidase in clinical isolates of MDR AB, so as to enhance the expression and activity of catalase peroxidase and weaken the killing effect of macrophages on MDR AB (Sato et al., 2019).Although macrophages play a small role in killing ABs, morphological changes occur in macrophages,a large number of pro-inflammatory factors, such as IL-6 and TNF-α, and neutrophil chemokine macrophage inflammatory protein-2 (MIP-2), are produced by macrophages within 4 h of infection and the loss of macrophages will greatly increase the bacterial load in the host, which indicates that after the host is infected with AB, macrophages are significantly activated and actively start innate immunity to deal with AB infection, which plays a vital role in controlling the development of AB infection (Lazaro-Diez et al., 2017; Qiu et al., 2012; Bruhn et al., 2015; Lázaro-Díez et al., 2017). After 24 h, the number of macrophages increased significantly at the site of infection (Qiu et al., 2012; Harris et al., 2019; Zeng et al., 2019). At the same time, macrophages act as antigen-presenting cells and increase the expression of the costimulatory ligands, CD80 and CD86 on their surface, thus initiating and regulating T cell immunity (Bhatia et al., 2006; Gu et al., 2021). Macrophages play direct and indirect roles in controlling AB infection and have an impact on subsequent immune responses. Hence, during the period from AB infection stimulating macrophages to start innate immunity to start specific immunity, it is the key to find AB infection and take corresponding measures to enhance innate immunity and eliminate AB to prevent further aggravation of infection. Whether AB infection can be detected through sharp rise of inflammatory factors such as IL-6 and TNF-α or the sharp rise of neutrophils in the alveoli or blood needs to be further verified.

## Response of Neutrophils in AB Infection

### Changes of Neutrophils in AB Infection

As the main innate immune cells to phagocytize and kill AB,further chemotaxis of neutrophils to the lung tissue after macrophage infiltration is an important factor in preventing the continued aggravation of AB infection (Van Faassen et al., 2007; Breslow et al., 2011). Zeng et al. (Zeng et al., 2019) inoculated the airways of mice with lethal SJZ24 AB and showed that BALF cells were dominated by alveolar macrophages, and neutrophils were hardly detectable before infection. The majority of BALF cells (80%) were neutrophils, 24 hours after infection. At the same time, Harris et al. (Harris et al., 2019) studied the intraperitoneal infection model of clinical isolate LAC-4 AB and found that neutrophils in the mouse abdominal cavity began to increase significantly 4 h after inoculation with LAC-4 AB and peaked within 24-48 h. The bacterial load in the abdominal cavity, blood, spleen, and kidney of mice decreased significantly after 24 h and was generally lower than the detection limit after 48 h; thus, they speculated that neutrophils may be important immune cells for clearing bacteria. A study by Lazaro-Diez et al. (Lazaro-Diez et al., 2017) verified this hypothesis. They cultured neutrophils and AB in human and bovine serum. They found that human neutrophils had taken up AB 3 h after infection, and come into contact with the bacteria, phagocytosed, and killed it within 4 h after infection. This study directly demonstrated the phagocytosis and killing effect of neutrophils on ABs (Lazaro-Diez et al., 2017). Bhuiyan et al. (Bhuiyan et al., 2016) found that selective depletion of zebrafish neutrophils increased susceptibility to acute lethal AB infection, while depletion of macrophages had no significant effect, which also showed that neutrophils were the main force for phagocytosis and elimination of bacteria in AB infection. Furthermore, gene expression profiling analysis of human neutrophils showed that the genes encoding the protein NFκB and the pro-inflammatory cytokines IL-6, IL-1β and CXCL-8 were overexpressed in human neutrophils after infection with AB *in vitro*, and the expression of NFκB enhanced the expression of its downstream pro-inflammatory factors. However, all of these overexpressed genes occurred after activation of the neutrophil TLR4 receptor by AB, so it is speculated that neutrophils may produce pro-inflammatory factors through the TLR4-NFκB pathway, which in turn attract other immune cells to the site of infection while mediating lung damage (Lázaro-Díez et al., 2020).If adopting artificial intervention measures makes the peak of neutrophil recruitment reached within 24 hours or even earlier after AB infection, the clearance rate of AB may be greatly increased, and even the activation of T cell immunity by macrophages may be avoided, so as to avoid lung injury caused by strong immune response.

### Mechanism of Neutrophil Recruitment

The primary mechanism of neutrophil chemotaxis to infection sites is macrophage recruitment, especially in the alveoli where macrophages account for almost 100% of cells in BALF (Qiu et al., 2012; Shapouri-Moghaddam et al., 2018; Harris et al., 2019; Zeng et al., 2019). After macrophages recognize the LPS or OMVs of AB through the TLR4 receptor, they produce neutrophil chemokines such as MIP-2, CXCL1/KC, and the inflammatory cytokine IL-6 through the TLR adaptor protein MyD88 pathway (De Filippo et al., 2008; Jhingran et al., 2015; Marion et al., 2019; Pires and Parker, 2019). In a study of neutrophil chemotaxis in mice by De Filippo et al. (De Filippo et al., 2008), it was found that the chemokine MIP-2 can also be produced through the adaptor protein TRIF pathway. Activation of NF-κB in alveolar macrophages leads to increased expression of IL-1β and TNF-α, and these early pro-inflammatory cytokines can also stimulate the production of chemokines such as cytokine-induced neutrophils chemokine 1 (Diep et al., 2018). These chemokines contribute to neutrophil recruitment to the site of infection through chemotactic migration. However, in macrophage-depleted mice, the reduction in AB clearance contradicts the increase in neutrophil recruitment (Lee et al., 2020), suggesting that macrophages are not the only mechanism of neutrophil recruitment to the infected site.

Mast cells are another important cell type that recruits neutrophils. Kikuchi-Ueda et al. (Kikuchi-Ueda et al., 2017; Kikuchi-Ueda et al., 2021) found that co-culture of human LAD2 mast cells with live AB for 4 hours *in vitro* can increase the migration of neutrophils, and lipooligosaccharide (LOS) derived from MDR AB stimulates LAD2 human mast cells to induce increased expression of pro-inflammatory mediators TNF-α, IL-8, LTC4S, and CCL4, mainly IL-8. Intact AB stimulants LAD2 human mast cells to mainly induce the production of proinflammatory mediator TNF-α. This shows that mast cells initiate the immune response against AB by releasing preformed mediators that activate effector neutrophils. MDR AB can induce mast cell to produce more IL-8 than that in standard strain, indicating that the structural difference in LOS of AB affects the immune regulation function of mast cells. Therefore, the structural analysis of LOS of MDR AB is worthy of further study to clarify the mechanism of LOS mediated mast cell immune response. In mouse intraperitoneal infection study and *in vitro* cell experiments, De Filippo et al. (De Filippo et al., 2013) found that under the stimulation of LPS, mast cells also released neutrophil chemokines, CXCL1 and CXCL2, through the TLR4-mediated MyD88 pathway, and in mast cell-deficient mice, LPS stimulation of the mouse peritoneum resulted in greatly reduced neutrophil recruitment to the site of infection. The difference between mast cells and macrophages is that after 24 h of LPS stimulation, macrophages also produce CXCL5 and CXCL7 to recruit neutrophils, whereas mast cells do not produce these two cytokines (De Filippo et al., 2013).

NK cells recruit neutrophils early in infection by increasing the expression of the chemokine, KC. Tsuchiya et al. (Tsuchiya et al., 2012) compared NK1.1+ cell-depleted mice with control mice and found that the levels of the chemokine CXCL1/KC in NK1.1+ cell-depleted mice during the first 3 days after infection with AB were lower, and the recruited neutrophils were significantly reduced.

The interaction of AB bacterial components with the host also affects the extent of neutrophil recruitment and local inflammatory responses. When AB OmpA and periplasmic protein TonB interact with airway epithelial cells, airway epithelial cells produce the antibacterial peptide LL-37. As an important neutrophil chemokine, this antibacterial peptide induces neutrophil recruitment to the infected site (Pires and Parker, 2019). The changes in AB bacterial membrane components also affect the immune response intensity of neutrophils to a certain extent. Overexpression of the structural genes of the AB AdeABC and AdeIJK efflux systems can generate MDR AB and lead to changes in associated cell membrane function, such as reduced biofilm. In a study of mouse nasal infection with AB mutants overexpressing *adeABC*, although the virulence of AB overexpressing *adeABC* strengthened, more neutrophils recruited to lung and more myeloperoxidase (MPO) was produced in BALF, which weakened its adaptability (survive capability or fitness) in the host. This study shows that overexpressing *adeABC* of AB increases the recruitment and activity of pulmonary neutrophils and the virulence of AB does not depend on its adaptability in the host (Yoon et al., 2016). Therefore, how *adeABC* overexpressed AB can increase its virulence and recruit more neutrophils through the changes of related molecules in the efflux system needs to be further explored. AB OMVs increase the release of chemokines in mouse lungs through interacting with macrophage TLR2 and TLR4 by MyD88 pathway or increasing mitogen-activated protein kinase (MAPK)-related phosphorylated P42/44 (or Erk1/2), thereby increasing neutrophil recruitment. Marion et al. (Marion et al., 2019) found that after intranasal administration of different doses of AB OMVs to mice, the concentrations of chemokine CCL2 and cytokines IL-6 and IL-1β in BALF increased in a dose-dependent manner. The number of neutrophils also increased in a dose-dependent manner and was significantly higher than that in the control group.

The metabolism of AB and its interaction with the host’s local environment also affect the immune responses of neutrophils. Neutrophil recruitment enhances the clearance of AB while inhibiting the AB phenylacetic acid catabolism pathway, resulting in the accumulation of the metabolic by-product phenylacetate. Phenylacetate is a bacterial-driven direct chemotactic agent that enables neutrophils to migrate rapidly to the site of infection and stay there (Bhuiyan et al., 2016). Rodman et al. (Rodman et al., 2019) found that the metabolism of AB is affected by the complex composition of pleural effusions. Induced by human pleural effusion, a phenylalanine (PA) catabolic pathway independent of the phenylacetic acid pathway that converts PA to phenylpyruvate (PP) is more likely to occur, and pleural effusion-induced PP degradation significantly reduces the chemotaxis of human neutrophils *in vitro*. The conversion of PA to PP and the degradation of PP induced by some components in pleural effusion needs to be further studied. Whether AB infection leads to the changes of some components in host pleural effusion and then affects the metabolism of AB also needs to be further explored.

### Mechanism of Neutrophils Scavenging AB

Neutrophils can control and eliminate ABs through oxygen bursts and the formation of neutrophil extracellular traps (NETs) (Chen, 2020). The major antibacterial mechanism of neutrophils is the oxygen burst, and NADPH oxidase is the major ROSproducer in phagocytes. Bacterial phagocytosis triggers an oxygen burst that causes the NADPH oxidase complex to assemble on the phagosomal membrane and produce superoxide. Superoxide dismutase converts superoxide into H_2_O_2_, which is then converted by MPO into a highly bactericidal hypochlorous acid that kills AB (Sun et al., 2016). The absence of NADPH oxidase hinders oxygen bursts, thus reducing the ability of neutrophils to scavenge AB. gp91^phox^ is the catalytic subunit of NADPH oxidase, and Qiu et al. (Qiu et al., 2009) found that the bacterial load of gp91^phox -/-^ mice inoculated with AB was significantly higher than that in wild-type mice. Although deletion of gp91^phox^ did not lead to a change in the number of neutrophils, it weakened the ability of neutrophils to kill AB. AB itself can also affect oxygen burst, which in turn affects neutrophil clearance. The *katG* and *katE* genes in AB can resist H_2_O_2_ and may enhance neutrophil oxygen burst. Sun et al. (Sun et al., 2016) showed that although Acinetobacter with *katG* and *katE* deletion were sensitive to H_2_O_2_, the deletion of both genes weakened the production of oxygen bursts in neutrophils, resulting in an increase in the mortality of the larval host infecting with A. nosocomialis. Although nosocomial bacteria with *katE* and/or *katG* were able to counteract the H_2_O_2_ produced by neutrophils, they induced a more intense oxygen burst, resulting in increased bacterial killing which needs to be further verified in AB. Whether the balance mechanism that *katE* and/or *katG* existence can resist H_2_O_2_ but induce more intense oxidative burst is related to the expression degree or proportion of *katE* and *katG* genes needs to be further studied at the levels of genomics, transcriptomics and proteomics. External factors such as alcohol can also weaken the bactericidal function of neutrophils by affecting the process of oxygen burst. Gandhi et al. (Gandhi et al., 2014) studied the effect of alcohol on killing AB by neutrophils. Alcohol affects the oxidative burst of neutrophils, resulting in the reduction of ROS produced by NADPH oxidase, thus reducing the killing of ABs by neutrophils.

Another antibacterial mechanism of neutrophils is the formation of NETs, which capture and kill the bacteria. NETs are unique DNA skeletons modified by antimicrobial peptides, which are formed by the release of their nuclear contents (unfolded chromatin and lysosomal enzymes) when neutrophils die (Kamoshida et al., 2018; Ravindran et al., 2019). Antimicrobial peptides (such as LL-37) are cationic antibacterial substances found in the lysosomes of neutrophils. Wild-type AB contains anionic LPS, and cationic LL-37 combines with anionic LPS to exert an antibacterial effect (Kamoshida et al., 2020). Autophagy is one of the mechanisms for NET formation (Boeltz et al., 2019; Douda et al., 2015; Germic et al., 2017). *In vitro* studies by Konstantinidis et al. (Konstantinidis et al., 2016) demonstrated that clarithromycin can act as an autophagy inducer for neutrophils, inducing the production of NETs containing the antimicrobial peptide LL-37 and inhibiting the growth and biofilm formation of AB. A previous clinical trial (Giamarellos-Bourboulis et al., 2008) also showed that the application of clarithromycin in AB VAP patients can accelerate the resolution of pneumonia and reduce the risk of death from septic shock, indicating that clarithromycin may act as an immune regulator *in vivo*, which induces NET formation, by increasing the clearance of MDR AB by neutrophils. The NET formation is also influenced by the interaction between relevant bacterial factors and neutrophil-associated receptors (Kamoshida et al., 2018). Lazaro-Diez et al. (Lazaro-Diez et al., 2017) observed in an *in vitro* experiment that neutrophils phagocytizing AB released fewer NETs than neutrophils directly stimulated by phorbol 12-myristate 13-acetate, a NET inducer. This phenomenon is consistent with the view of Kamoshida et al. (Kamoshida et al., 2018) that ABs inhibit the production of NETs, thereby escaping killing by neutrophils to a certain extent. Kamoshida et al. (Kamoshida et al., 2018) also demonstrated that AB inhibits neutrophil adhesion by inhibiting the expression of the neutrophil surface molecule CD11a, thereby affecting NET production. Studies of the specific component of ABs interacting with specific receptors on neutrophils through signaling pathways to inhibit the production of NETs should be further studied as a component in the innate immunotherapy of MDR ABs. In addition, in the mechanism of clarithromycin induced NET formation, whether there is a pathway to increase the expression of CD11a in neutrophils to resist the reduction of NET formation caused by AB inhibiting CD11a formation is worthy of further exploration (**Table 1**).

**Table 1:** Neutrophil and macrophage response in Acinetobacter baumannii infection.

Research object	Effector	Effect	Ending	reference
OMV	Macrophage	TLR2,TLR4-MyD88/MAPK— CCL2,CXCL1,IL-6,IL-1β,TNF-α	Neutrophil recruitment, killing of AB, lung injury	De Filippo et al., 2008; Jhingran et al., 2015; Marion et al., 2019; Pires and Parker, 2019,
LPS	Macrophage	TLR4-MyD88/TRIF/NFκB— IL-6,TNF-α,MIP-2,CXCL1/KC, IL-1β	Neutrophil recruitment, killing of AB, lung injury	De Filippo et al., 2008; Jhingran et al., 2015; Diep et al., 2018; Marion et al., 2019; Pires and Parker, 2019
OmpA/TonB	Airway epithelium	LL-37	Neutrophil recruitment, killing of AB, lung injury	Pires and Parker, 2019
LOS	Mast cells	TLR4-MyD88—CXCL1,CXCL2, IL-8, TNF-α,LTC4S,CCL4	Neutrophil recruitment, killing of AB	De Filippo et al., 2013
AB	NK cell/DAMPs	KC/NLRP3-ASC-caspase1/11- IL-1β	Neutrophil recruitment, killing of AB, lung injury	Tsuchiya et al., 2012; Kang et al., 2017; Dikshit et al., 2018; Xiong et al., 2020

AB, Acinetobacter baumannii; OmpA, Outer membrane protein A; OMVs, outer membrane vesicles; LPS, lipopolysaccharide; TLR, Toll-like receptor; MIP-2, macrophage inflammatory protein-2; LOS, Lipooligosaccharide.

## Lung Injury

AB can cause multiple organ, bloodstream, and nosocomial infections, especially VAP, which are the most common types of diseases caused by AB infection. As important pathogenic bacteria, AB is mostly MDR and difficult to treat (Martin-Aspas et al., 2018; Chen, 2020). The immune response triggered by AB infection is a major cause of lung injury. Innate immunity is the first line of defense against infection. In the face of AB infection, the impact of immune effects on lung injury is worth studying, especially the impact of the immune effects of macrophages and neutrophils.

The interaction between AB bacterial components and the host affects the degree of lung injury. Marion et al. (Marion et al., 2019) introduced AB OMVs into the nasal cavity of mice to mediate pulmonary inflammation and found that the interaction of OMVs with mouse alveolar macrophage TLR2 and TLR4 could increase the production of the chemokines, CCL2 and CXCL1, and the pro-inflammatory cytokines, IL-6, IL-1β, and TNF-α in the mouse lungs, which led to a massive influx of neutrophils into the alveoli and mediated alveolar epithelial damage and airway obstruction, leading to lung consolidation. Compared with wild-type mice, the inflammatory response in the lung was inhibited in TLR2- and TLR4-deficient mice after OMVs stimulated the nasal cavity, and the degree of lung consolidation was greatly reduced. The interaction between OMVs, TLR2, and TLR4 promotes AB-mediated lung injury. In addition, AB OMVs carrying OmpA invading lung epithelium can mediate its accumulation on mitochondria by activating the GTPase dynamin-related protein 1 (DRP1), resulting in mitochondrial fragmentation, increased ROS release and cell death. Meanwhile, OmpA carried by OMVs can also mediate mitochondrial lysis and cell death in macrophages. The death of lung epithelial cells and lung macrophages leads to the breakdown of the innate immune barrier in the lung, aggravating lung injury and the spread of infection (Tiku et al., 2021).The application of polymyxin induces the production of polymyxin-resistant LPS-deficient AB clinical isolates (Moffatt et al., 2010; Lean et al., 2014; Girardello et al., 2017; Carretero-Ledesma et al., 2018). LPS-deficient AB infections in patients with neutropenia or immunodeficiency may lead to severe lung injury. Kamoshida et al. (Kamoshida et al., 2020) showed that LPS-deficient AB can only weakly stimulate neutrophils and produce a small amount of ROS and inflammatory cytokines but is sensitive to lysozyme produced by neutrophils. Therefore, LPS-deficient AB was still destroyed by neutrophils. However, in patients with neutropenia or immunodeficiency, LPS-deficient AB may reside in the lung for a long time owing to insufficient lysozyme production, aggravating the degree of lung injury.

High expression of IL-1β can cause acute lung injury (Diep et al., 2018). IL-1 β binding to the receptor IL-1R1 in endothelial cells reduces the activity of soluble adenylate cyclase, resulting in the production of cAMP and the down-regulation of the expression of transcription factor cAMP response element binding, which reduces the expression of VE cadherin (the core component of endothelial adhesion junction) and promotes the loss of VE cadherin junction integrity leading to pulmonary vascular leakage and acute inflammatory lung injury (Xiong et al., 2020). The inflammasome NLRP3-ASC-caspase1/11 complex is required for IL-1β production by macrophages. AB initiates and activates the NLRP3 inflammasome in macrophages through damage-associated molecular patterns, such as extracellular ATP, K^+^ efflux, production of ROS, and release of cathepsins. After that, NLRP3 inflammasome activates its downstream molecular adaptor protein ASC, which in turn recruits and activates caspase-1, enabling macrophages to produce IL-1β and promote the maturation of IL-1β, resulting in lung injury (Kang et al., 2017; Dikshit et al., 2018). IL-1β is also a chemokine for neutrophils, and the inflammasome NLRP3 can recruit neutrophils to the lung through IL-1β in the late stage of AB infection (Dikshit et al., 2018). At the same time, neutrophils can also produce IL-1β through the inflammasome NLRP3 pathway, which further aggravates lung injury (Kang et al., 2017; Dikshit et al., 2018). In addition to the production of IL-1β through the NLRP3 inflammasome to mediate lung injury, neutrophils may also mediate the high expression of IL-1β through the TLR4-NFκB pathway, causing lung injury (Lázaro-Díez et al., 2020). While demonstrating that AB induces macrophages to produce IL-1β through the NLRP3 inflammasome pathway to mediate lung injury, Kang et al. (Kang et al., 2017) used *in vitro* cell experiments to demonstrate that AB may mediate pyroptosis of macrophages through caspase11, and Dikshit et al. (Dikshit et al., 2018) demonstrated that AB mediates the production of IL-1β and IL-1α, a related cytokine to IL-1β, through caspase11 in animal experiments and pyroptosis of macrophages and lung injury which displays a probable relationship between IL-1α and pyroptosis of macrophages and lung injury but the direct mechanism is unclear. Monitoring levels of IL-1α and IL-1β in alveoli or blood in the early stage of AB infection and taking some measures to reduce their production may alleviate the degree of lung injury and improve the prognosis.

The absence of macrophages can lead to lung damage. Functionally, M2 macrophages, which can secrete anti-inflammatory factors IL-10 and TGF-β, have strong phagocytosis ability, can remove debris and apoptotic cells, promote tissue repair and wound healing, and can promote angiogenesis and fibrosis (Shapouri-Moghaddam et al., 2018). The loss of macrophages, especially M2 macrophages, will lead to the increase of AB load in the lung, resulting in a large number of neutrophils pouring into the infected site through other mechanisms. A large number of phagocytes and apoptotic neutrophils cannot be cleared in time, and newly stimulated neutrophils continue to flow into the infected respiratory tissue, which can lead to the harmful residence of leukocytes and extensive alveolar-capillary damage (Lee et al., 2020). The polarization of macrophages to M2 type is finished by Th2 cytokine IL-4 and IL-13 through IL-4 receptor α activating STAT6 pathway or IL-10 through IL-10 receptor activating STAT3 pathway (Shapouri-Moghaddam et al., 2018). Meanwhile, IL-10 activates STAT3 pathway through IL-10 receptor mediating the expression of MARCO (scavenger receptor on the surface of macrophages) to further mediate phagocytosis and killing functions of macrophage (Kang et al., 2020).Choosing an appropriate time to give macrophage polarization promoting factors or its corresponding receptor activators to the host may increase the clearance of AB and dead phagocytes, improving the harmful residence of leukocytes and extensive alveolar capillary damage caused by the lack of M2 macrophages. Another study showed that in female mice, the depletion of macrophages affects the protein and mitochondrial metabolic functions of mice infected with AB, which in turn leads to the disruption of metabolic homeostasis in infected mice and the phagocytic function of macrophages,which may make lung damage worsen (Tsotakos et al., 2016; Pires et al., 2020).

As the main force of phagocytosis and elimination of bacteria in AB infection, neutropenia also aggravates lung injury (Van Faassen et al., 2007; Breslow et al., 2011). Several studies have shown that after infection with AB, neutrophils can be rapidly recruited in the lung within 4 hours, and reach a peak at 24 hours, so that quickly engulf and kill AB in the lungs,which greatly reduce the bacterial load in the lungs and inhibit the spread of bacteria outside the lungs (Van Faassen et al., 2007; Lázaro-Díez et al., 2017; Harris et al., 2019; Zeng et al., 2019). The absence of neutrophils not only reduces the clearance of AB, but also delays the secretion of various inflammatory factors and chemokines, such as TNF-α, IL-6, IL-10 and MCP-1,leading to further increased bacterial load in the lungs. The aggravated bacterial load and the extrapulmonary spread of bacteria further induce the production of a large number of pro-inflammatory factors, which mediate pulmonary epithelial mucosal damage and fibrinous exudation. Lots of bacteria, necrotic cell debris, and inflammatory exudate accumulate in the alveoli, causing lung consolidation (Van Faassen et al., 2007). Polymyxin-resistant LPS-deficient AB may reside in the lung for a long time due to insufficient production of neutrophil lysozyme, aggravating the degree of lung injury (Kamoshida et al., 2020). Therefore, administration of neutrophil chemokines or proinflammatory cytokines in neutropenic patients to increase intrapulmonary recruitment of neutrophils early in infection may help attenuate lung injury.

The nucleotide-binding oligomerization domain (NOD)-like protein NOD2 is an intracellular pathogen recognition receptor that senses the peptidoglycan components of the bacterial cell wall. Kale et al. (Kale et al., 2017) found that NOD2^−/−^ mice had significantly reduced pulmonary ROS/reactive nitrogen species (RNS) production and increased pulmonary bacterial load 4 hours after AB infection, indicating that NOD2 can promote early defense against AB infection by mediating ROS/RNS production, and early clearance of AB helps reduce lung damage from subsequent immune responses. RIP2 is an adaptor molecule that plays a role in signal transduction in the NOD2-mediated intracellular antibacterial response pathways. Silencing RIP2 affects the activation of NF-κB in lung epithelial cells and reduces the production of TNF-α and IL-8 (Bist et al., 2014). The decrease in TNF-α and IL-8 levels increases the invasion of epithelial cells by AB. AB interacts with the airway epithelium to produce the antimicrobial peptide LL-37 (neutrophil chemokine), resulting in increased neutrophil recruitment and lung injury (Kale et al., 2017; Pires and Parker, 2019).

Among the factors causing host lung injury caused by infection with AB, the effect of host gender difference on lung injury is based on the number of immune cells *in vivo* after infection (Pires et al., 2020). Studies have shown that in female mice infected with AB, both macrophages and neutrophils in the airways are reduced, and the degree of inflammation is increased. Estrogen can not only affect the clearance of AB and aggravate lung injury by reducing the number of immune cells, blocking the migration of neutrophils, and reducing the production of ROS, but also promote the metabolism of iron in the body to a certain extent (however, exogenous estrogen will inhibit the estrogen response element in the hepcidin promoter, thereby inhibiting iron metabolism), which may further promote the uptake of iron by AB and prolong the survival of AB, resulting in increased lung damage. And male mice receiving exogenous estrogen injection may increase the expression of the siderophore acinetobactin due to the inhibition of iron metabolism, so that the acinetobactin-mediated epithelial cell apoptosis will also aggravate the lung damage caused by AB (Li et al., 2018; Pires et al., 2020).

Mechanical ventilation can also cause lung injury. A study by Tsay et al. (Tsay et al., 2021) has shown that mechanical ventilation after infection with AB reduces the bactericidal activity of alveolar macrophages by reducing the production of TNF-α, thereby enhancing AB-induced lung injury. Simultaneously, mechanical ventilation increases the expression of vascular cell adhesion molecule, MIP-2, and IL-6 in the lungs through the JNK1 signaling pathway and increases the levels of inflammatory cells and nitrite in the alveoli, leading to lung injury. Whether mechanical ventilation causing lung injury through JNK1 pathway is related to affecting lung epithelial cells through the changes of ventilator parameters (airway pressure, oxygen concentration and other physical factors), or causing infections in lung epithelial cells through pathogens in ventilator needs to be further studied (**Table 2**).

**Table 2:** Mechanism of lung injury.

Location	Medium	Mechanism	Ending	Reference
Pulmonary vascular endothelium	IL-1β	IL-1β—IL-1R1—cAMP decrease—VE cadherin decrease	pulmonary vascular leakage and acute inflammatory lung injury	Xiong et al., 2020
Pulmonary epithelium	DRP1	OmpA—DRP1 activate—mitochondrial fragmentation	increased ROS release, death of lung epithelial cells and lung macrophages	Tiku et al., 2021
	acinetobactin	Exogenous estrogen— Iron metabolism inhibition—acinetobactin of AB increase	Induce epithelium cell apoptosis, pulmonary epithelium injury	Li et al., 2018; Pires et al., 2020
Airway obstruction	Inflammatory cell and cytokines; Neutrophil recruiment	Macrophage depletion— harmful residence of leukocytes and extensive alveolar capillary damage; neutrophil depletion—Inflammatory cytokines secretion delayed—bacteria load increase—inflammatory factors burst; NOD2^-/-^ –ROS/RNS decrease—bacteria load increase; RIP2 silence—NFκB anactivated—TNF-α,IL-8 decreased—LL-37 increased—neutrophil recruitment; Endogenous estrogen— Iron metabolism promotion— AB long life; Mechanical ventilation— JNK1—VACM,MIP-2,IL-6 increase—nitrite, inflammatory cells increase	pulmonary epithelial mucosal damage and fibrinous exudation; bacteria, necrotic cell debris, and inflammatory exudate accumulate in the alveoli;lung consolidation	Lee et al., 2020; Van Faassen et al., 2007, Bist et al., 2014, Kale et al., 2017; Li et al., 2018; Pires and Parker, 2019; Pires et al., 2020; Tsay et al., 2021
Exudation				
Exudation				

AB, Acinetobacter baumannii; TLR, Toll-like receptor; ROS, reactive oxygen species; NOD, nucleotide-binding oligomerization domain; RNS, reactive nitrogen species; DRP1, GTPase dynamin-related protein 1.

## Prospects of Immunotherapy

As mentioned above, inducing the body to produce specific adaptive immunity against AB is not suitable for patients to fight the infection immediately, and the timing and mechanism of intervention to fight infection in the early stage of infection with innate immunity should be considered (Gu et al., 2021).

The introduction of trained innate immunity makes it possible for vaccines to induce rapid innate immune responses independent of adaptive immune memory. A related study of Gu et al. (Gu et al., 2021) showed that alveolar macrophages immunized with AB inactivated whole cell undergo immune activation (2 days after vaccination), immune quiescence, immune memory (5 days after vaccination) and epigenetic, metabolic, and functional reprogramming that can produce more TNF-α by upregulating TLR4 in alveolar macrophages. As a hallmark functional cytokine of trained innate immunity, TNF-α further activates downstream cytokine responses and immune cells, and further stimulates macrophages to produce immune memory, which can resist the secondary invasion of AB more quickly (Gu et al., 2021). There is currently uncertainty about how long the effects of trained innate immunity will last, and in order to make vaccines more effective, it may be a good idea to integrate trained innate vaccines and adaptive immunity vaccines. Clarithromycin promotes NET formation by inducing neutrophil autophagy, which in turn promotes neutrophil to capture and kill AB (Konstantinidis et al., 2016). A previous clinical study of Giamarellos-Bourboulis et al. (Giamarellos-Bourboulis et al., 2008) has shown that intravenous application of clarithromycin for 3 days can help resolve pneumonia in patients with AB VAP, thus confirming that clarithromycin has an immunomodulatory effect *in vivo*. However, the specific mechanism by which clarithromycin induces neutrophils to generate NETs *in vivo* and whether the combination with other antibiotics affects the generation of NETs needs further study. The mechanism by which AB inhibits the production of NETs to protect itself from being killed by neutrophil NETs also needs further studies (Kamoshida et al., 2018).

Enhanced clearance of AB by inducing increased neutrophil recruitment could also be a direction for innate immunotherapy against AB infection. Chen (Chen, 2020) proposed that administration of MIP-2 or immunomodulator 3’-5’-cyclic dimer guanosine monophosphate to mice with AB infection increased the aggregation of local neutrophils in mice, thereby enhancing the clearance of AB. However, Kale et al. (Kale et al., 2017) showed that the loss of NOD2 leads to a weakened lung defense against AB in the early stage of AB infection. The increase in neutrophil recruitment in the lungs and aggravation of lung inflammation contradicted the conclusion of the above study that stated that enhancing the clearance of AB can be achieved by increasing the recruitment of neutrophils, indicating that the effect of neutrophil clearance of AB may be mediated by NOD2. At the same time, they also confirmed that intraperitoneal administration of the NOD2 ligand muramyl dipeptide after infection with AB protected mice from AB infection in the lung (Kale et al., 2017). Whether the combined application of neutrophil chemokines and NOD2 ligands can play a more prominent role in the early eradication of AB should be further studied.

Except enhance the clearance of MDR AB by directly enhancing the innate immune response, indirectly enhancing the innate immune effect by targeting the bacterial components of AB to interfere with its mechanisms of pathogenesis and escaping from innate immunity may also be helpful to solve the problem of drug resistance. The AB capsule is a worthy target. AB capsule is not only related to the functions of bacterial cell adhesion, invasion and biofilm formation, but also can resist the phagocytosis of phagocytes (Niu et al., 2020). The presence of the AB capsule will hinder the release of LPS, further hinder the phagocytosis of AB by macrophages, and limit the production of pro-inflammatory cytokines and chemokines such as IL-6, G-CSF, and MIP2, so that AB can escape the killing and phagocytosis effect of the body’s innate immunity and survive. Targeted lysis of the capsule may help activate innate immune cell TLR4-TRIF-IRF3 pathway-mediated phagocytosis and enhance bacterial clearance from the body (Akoolo et al., 2022).

As mentioned above, the differences in immune responses and metabolic levels caused by gender differences have an impact on the outcome of AB infection. For example, in mice with pulmonary infection with Pseudomonas aeruginosa, lactoferrin expression decreased in male mice treated with estrogen (Wang et al., 2010). Lactoferrin as an iron chelating antibacterial agent, its reduction will increase the body’s free iron, and iron ion is very important for the survival and pathogenesis of AB. The use of estrogen may promote the reproduction of AB and aggravate infection and lung injury. An in-depth understanding of the impact mechanism of gender differences in the interaction between the host and AB can help patients receiving hormone therapy to rationally design immune modulation strategies (Li et al., 2018; Pires et al., 2020).

## Conclusion

The roles of neutrophils and macrophages in innate immunity are vital for fighting AB infections. Studying the relationship between the degree of neutrophil and macrophage responses and lung injury in AB infection is beneficial for the development of new immune strategies for controlling the infection in the early stages and reducing the degree of lung injury. At present, research on the relationship between the lung innate immune response state and lung injury needs to be further explored, which may help solve the global public health problem of antibiotic resistance and promote a benign prognosis for AB infection.

## Author Contributions

ZL drafted and revised the manuscript and WX reviewed and revised the manuscript for publication. All authors contributed to the article and approved the submitted version.

## Funding

This work was supported by Key Research and Development Guidance program of Liaoning Provincial (No. 2019JH8/10300023).

## Conflict of Interest

The research was conducted in the absence of any commercial or financial relationships that could be construed as a potential conflict of interest.

## Publisher’s Note

All claims expressed in this article are solely those of the authors and do not necessarily represent those of their affiliated organizations, or those of the publisher, the editors and the reviewers. Any product that may be evaluated in this article, or claim that may be made by its manufacturer, is not guaranteed or endorsed by the publisher.

## Abbreviations

AB, Acinetobacter baumannii; MDR, multidrug resistance; VAP,ventilator-associated pneumonia; OmpA, Outer membrane protein A; OMVs, outer membrane vesicles; LPS, lipopolysaccharide; BALF, bronchoalveolar lavage fluid; TLR, Toll-like receptor; MIP-2, macrophage inflammatory protein-2; LOS, Lipooligosaccharide; MPO, myeloperoxidase; PA, phenylalanine; PP, phenylpyruvate; NETs, neutrophil extracellular traps; ROS, reactive oxygen species; NOD, nucleotide-binding oligomerization domain; RNS, reactive nitrogen species; DRP1, GTPase dynamin-related protein 1.
